# 

**Published:** 1877-04

**Authors:** 


### Contents of April Number.

### ORIGINAL.

ART. I.—Address of Prof. E. V. Stoddard, M. D., to the Graduating Class of the Buffalo Medical College, February 20th, 1877...... 203
ART. II.—The Hip Joint, by E. R. Barnes, read before the Buffalo Medical Association,........ ... .. ... .................... 217

### MISCELLANEOUS.

Accidental loss of Drainage-tubes in Pleural Cavity.—Removal by Operation,...................................................... 226
Tetanus or Hydrophobia.—Letter to Dr. E. S. Gaillard................. 228
Aspiration in Treatment of Hernia,................................ ... 229
Anæsthsia by injection of Chloral into theVeins,...................... 230
A Case of Hydrophobia,......................... ................ 231

### EDITORIAL.

The Situation,............. ................................... 233
The Bergman vs. "Dr." Volker Case Settled,......................... 234
Changes in Drug Trade,.................... ...................... 235
BOOKS REVIEWED:
A series of American Clinical Lectures,......................... 236
The Electric Bath,... .......... ....................... 239
Spiritualism and Allied Causes and Conditions of Nervous Derangement,........................ ................ 241
Books and Pamphlets Received,................................ 241

